# Reducing short-acting beta-agonist overprescribing in general practice: Evaluation of a quality improvement programme in East London

**DOI:** 10.1080/13814788.2026.2619229

**Published:** 2026-02-02

**Authors:** Anna De Simoni, Hajar Hajmohammadi, Paul Pfeffer, Jim Cole, Chris J Griffiths, Sally A. Hull

**Affiliations:** ^a^ Wolfson Institute of Population Health, Centre for Applied Respiratory Research, Innovation, and Impact (CARRii), Queen Mary University of London, London, UK; ^b^ Department of Respiratory Medicine, Barts Health NHS Trust, London, UK

**Keywords:** Primary care, asthma, prescribing

## Abstract

**Background:**

Overprescribing of short-acting beta-agonist (SABA) inhalers is a worldwide problem.

**Objectives:**

To evaluate the impact of a system-wide quality improvement programme on SABA overprescribing, and to identify the most effective strategies.

**Methods:**

All general practices within one East London borough received the intervention between October 2020 and March 2023. Practices in two neighbouring boroughs acted as comparators. Intervention practices engaged in quality improvement activities including: electronic alerts flagging patients prescribed ≥12 SABA inhalers/year; generating lists of patients overprescribed SABA to call for review; a summary guideline for clinicians; electronic patient information leaflets. All practices were offered webinar coaching. Prescribing data were collected from electronic health records, and SABA overprescription evaluated through interrupted times series analysis. Content analysis was applied to survey data and conversations with staff.

**Results:**

During the three-year study period all localities introduced programmes to reduce SABA prescribing. We observed a significant decrease in the proportion of asthma patients prescribed more than 6 SABA/year in the study practices. The COVID pandemic triggered a temporary increase in patients on asthma registers, which persisted for 6 months. When implemented by practices the electronic prescribing alerts were effective: 50% of patients who received an active response reduced to <12 SABA in the following year.

**Conclusions:**

This quality improvement programme was associated with a reduction in SABA overuse, which could also decrease hospital admissions. Practices required individual coaching to use the electronic tools effectively. Integrated prescribing alerts reduced overprescribing, and collaborative practice cultures supported faster implementation of improvement strategies.

## KEY MESSAGES

- Using a learning health system built on electronic prescription data from primary care was associated with a reduction in short-acting beta-agonist (SABA) overprescribing.
- Prescribing alerts, integrated within the practice software, were associated with a reduction in overprescribing in the subsequent year.
- Localities which had a culture of general practices working together were able to implement quality improvement and other strategies more rapidly.

## Introduction

The dangers of SABA over-prescribing have been highlighted in national (BTS, NRAD) and international (GINA) guidance for many years [1]. However, SABA overprescribing remains common across healthcare systems as highlighted in the recent SABINA studies [2]. Notably, SABA overprescribing is more common in the UK than other European countries [2]. Recent data show that SABA overuse (defined as collection of more than two SABA canisters/year) is associated with a dose-dependent increased risk of all-cause mortality, increased use of antidepressants, hypnotics and sedatives, suggesting that those overprescribed SABA are a frailer patient group [3]. Another study found that SABA overuse (≥3 prescribed inhalers/year) was prevalent across all GINA steps, which may indicate suboptimal asthma control [4], and concludes that further studies need to investigate effective interventions to reduce it.

In addition to the patient harms associated with excess prescription of SABA, pMDIs (pressurized Metered-Dose Inhalers) are a major contributor to healthcare associated carbon footprint, and thereby global warming [5,6].

In East London, hospitalisation for acute asthma is 14% above the average for London, with hospital admissions rising from 1.3 to 7.5 per 100 asthma population as the number of SABA inhalers prescribed rises from 1to 3 to more than 12 a year [7]. As an estimate, by enabling a reduction of SABA prescribing from ≥12 SABA inhalers/year to between 4 and 12 SABAs (associated with a hospital admission rate of 2.3% a year), there is potential to avoid up to 70% of hospital admissions in this group [7].

Rates of SABA over-prescribing remain high in this multiethnic, deprived urban population, with an average >30% patients prescribed ≥6 SABA inhalers in the previous year, with significant variation between practices [8,9]. Among adult patients prescribed between 6 and 12 SABA inhalers/year, over a quarter of patients were issued less ICS prescriptions than expected [7]. Working with these patients to improve regular preventer use should be an early target to reduce SABA over-prescribing, by improving asthma control and reducing breakthrough asthma symptoms requiring reliever medication [10,11].

This quality improvement programme aimed to enable primary care clinicians to focus management attention on patients with asthma who were at highest risk of hospital admission. To achieve this a learning health system approach was used. The key to this is the use of real time clinical data which *‘turns data into knowledge, and knowledge into practice’* [12]. It aims to learn from every patient contact using the data for both clinical improvement and to improve the delivery system for improved population health. Prescribing data from general practice is well suited to this approach. We analysed asthma prescribing before and after implementing a Quality Improvement (QI) initiative aimed at limiting SABA overprescription.

## Methods

### Setting

The QI project took place in East London between October 2020 and March 2023, throughout the period of the COVID-19 pandemic. The intervention group included all 48 practice teams, with a registered population of approximately 400,000 people, belonging to 10 Primary Care Networks (PCNs) in NHS Newham borough, London, UK. The neighbouring boroughs of City & Hackney (39 GP practices) and Havering (45 GP practices) were used as natural controls. The intervention and control boroughs were chosen after ascertaining an absence of asthma improvement programmes at the start of the QI period.

In the 2021 UK census, 55% of the population in these boroughs were recorded as being of non-white ethnic origin [13], and the English indices of deprivation 2015 show that these localities fall into the top decile of the most socially deprived boroughs in England [14].

### Study population

The study population included all patients, aged 5–80 years, with a coded diagnosis of asthma and at least one prescription for inhaled asthma medication in the previous year. Study patients were required to be registered at the practice for at least one year prior to data extraction. All practices use the EMIS Web clinical system.

This study used an open cohort design using patient data from all 50 practices in Newham (and similar patient data from all 84 practices in the control boroughs).

### Quality improvement intervention

The QI programme was part of REAL HEALTH, a programme grant funded by Barts Charity. The programme was endorsed by the respiratory lead for Newham and by the commissioning organisation, but did not receive financial incentivisation.

The intervention included:

- In-consultation prescribing alerts for patients using >6 SABA inhalers in the previous six months. The pop-up alert tool displays at the centre of the screen, hence a response has to be made before proceeding. The alert is designed to open when the medical record is accessed, triggers only once/day and contains a choice of 5 coded responses:
- verbal invitation for an asthma review
- send SMS message or letter for patient to arrange an appointment for review (with SMS text and letter templates accessible though EMIS)
- medication review completed with the patient
- cancel the alert for the day
- permanently cancel the alert (clinicians only).
- Patient lists, based on real time prescribing data, identifying patients using high numbers of SABA and low numbers of ICS inhalers in the previous year. The lists can be used for recall by the nurse, pharmacist or GP.
- Provision of clinician summary guidance (See Supplementary material 1) based on current evidence and national guidelines, distributed at regular intervals as a hard copy to all GPs in Newham, and as electronic copy by email. This also included a one-page sheet for clinicians on topics to cover during the clinical review. Summary guidance and one-page sheet for clinicians to guide asthma reviews were posted once in print, followed by 18 email rounds at regular intervals.
- A patient information leaflet integrated within the asthma review template in EMIS. The leaflet could be distributed to patients during asthma reviews as printed or electronic copy via SMS. These resources were also available on the QMUL REAL HEALTH programme webpage.
- Web-based education webinars for clinicians to support the effective use of the IT tools and nurse-led asthma-reviews, including online advice for patients. The webinar was also summarised in a 5 minute YouTube video [15], with the link shared during the webinars, together with a SurveyMonkey feedback form [16]. Feedback received was used to update the QI activities and subsequent webinars.
- Endorsement by local respiratory leaders, practice networks and commissioners to ensure rollout of the intervention to all practices.
- Structured management during asthma reviews by first assessing the need for asthma education or adherence support and offering appropriate educational resources. Patients overusing SABA and not on ICS were to be started on ICS, while those on low-dose ICS were advised to step up treatment. Individuals overusing SABA despite high-dose ICS were to be considered for referral to secondary care and potential treatment escalation. The guidance outlined specific actions based on SABA use and ICS dosage, including initiating ICS for those not on controller therapy, adding a LABA for patients on low-dose ICS, adjusting LABA or increasing ICS depending on response, and considering additional therapies such as leukotriene receptor antagonists or theophylline if control remained inadequate (see page 5–6 in Supplementary material 1).

### Data sources

Asthma prescribing data was extracted monthly from EMIS Web for intervention and control boroughs, starting in October 2019 and continuing until the end of March 2023.

Each QI tool response was linked to an EMIS code – hence it was possible to quantify tool use and stratify responses by practice staff role.

During webinars, written notes were taken of all discussions about SABA prescribing. Healthcare professionals’ feedback was collected *via* SurveyMonkey feedback form [16].

### Data analysis

The analysis used interrupted time series methodology with monthly prescribing extracts going back 12 months prior to each point in time. The borough was the unit of analysis. Interrupted Time Series (ITS) Analysis is a quasi-experimental design used to assess the impact of an intervention by comparing data collected at multiple points before and after the intervention [17]. The ‘interruption’ marks when the intervention occurs, allowing to observe any significant changes in the trend or level of the outcome over time.

Analysis was undertaken in R (version 4.2.0).

The primary outcome was the change in % of patients showing SABA overuse; defined as patients who were prescribed 12 or more SABA inhalers in the previous 12 months.

Secondary outcomes were the change in % of patients showing SABA overuse defined as 6 or more SABA in the previous 12 months; the proportion of asthma patients prescribed inhaled corticosteroids (ICS) and the use of in-consultation prescribing alerts by practices, and use by different staff members.

A multi-group interrupted time series analysis (ITSA) model was developed, using Newham (NH, the intervention borough), and the average of the comparison localities, City & Hackney (CH), and Havering (Hv) before and after implementation of the QI project. The model was run based on 3 outcomes: (1) %SABA > 12, (2) %SABA > 6 and (3) %ICS/asthma patients.

The reduction in hospital admissions was estimated using data from the London borough of Newham reported in a previous study conducted in East London. That study described the proportion of hospital admissions among patients with asthma who were overprescribed SABA a few years prior to the QI. These proportions were applied to the Newham population to estimate hospital admissions before and after the QI intervention, and the difference between these estimates was taken as the projected reduction in admissions.

Written notes based on discussion with audience members during webinars were analysed using content analysis.

No attempt was made to isolate the impact of each individual activity or analyse demographic characteristics in this study. The baseline characteristics of the study population, including local SABA and ICS prescribing patterns, were reported in the baseline assessment study [8].

## Results

Data from the Electronic Health Records (EHRs) for all practices in the intervention borough Newham and the control boroughs (City&Hackney and Havering) were available for analysis.

**Figure 1.**
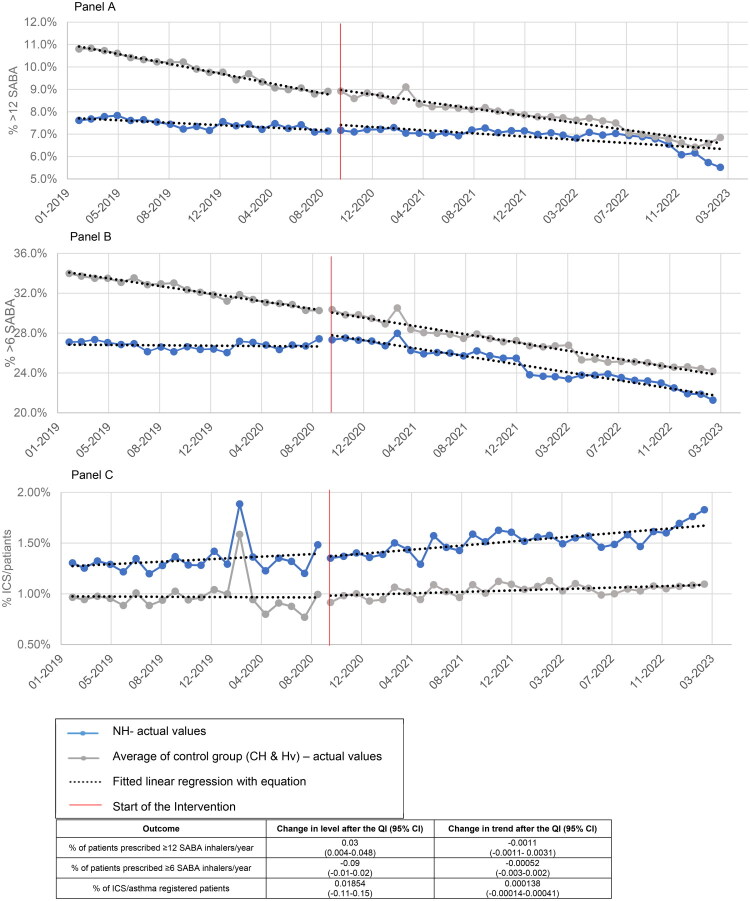
Multi-group interrupted time series analysis (ITSA) for 3 outcomes: Panel A) SABA >12, Panel B) SABA >6 and Panel C) ICS. Blue line is for NH and Grey line is for the average of CH and Hv (control group). The results of the interrupted time series analysis for the three boroughs are presented at the bottom.

The characteristics of the asthma study population have been reported previously [9]. During the period of the QI, all practices (both intervention and control) in the study area undertook a range of incentivised activities connected with limiting excessive SABA prescribing (see Box 1). A trend towards a decrease in SABA overprescribing was observed in all boroughs between January 2019 and October 2020, prior to commencing the QI intervention (Figure 1).

### Asthma inhaler prescribing before and after the intervention

The mean percentage of patients prescribed ≥12 SABA inhalers/year across all practices was 7.2% in Newham, 7.6% in City and Hackney and 9.6% in Havering boroughs, decreasing to 5.5%, 5.3% and 8.5%, respectively, by the end of the QI (Figure S1, panel A) At the start of the QI in October 2020 compared to the end of the QI in March 2023, the mean figures for patients prescribed ≥6 SABA inhalers/year were 27% in Newham, 29% in City and Hackney and 32% in Havering boroughs. This decreased to 21%, 23% and 25% respectively by the end of the QI (*p* = 0.000, Figure S1, Panel B).

For prescribing ≥12 SABA, The ITS results for the intervention borough (Newham) indicate that level change is significant (Figure 1, Panel A, *p* value = 0.019) but not its slope. That means the decrease in prescribing ≥12 SABAat the start of the intervention was significant, but not the trend after that. The ITS results show that trend (slope) and levels for SABA ≥ 6 in NH (before and after QI) were statistically significant, with *p* value =0.000. (Figure 1, panel B). The number of ICS prescriptions increased in Newham borough compared to control boroughs, though this was not statistically significant (Figure 1, Panel C).

**Figure 2.**
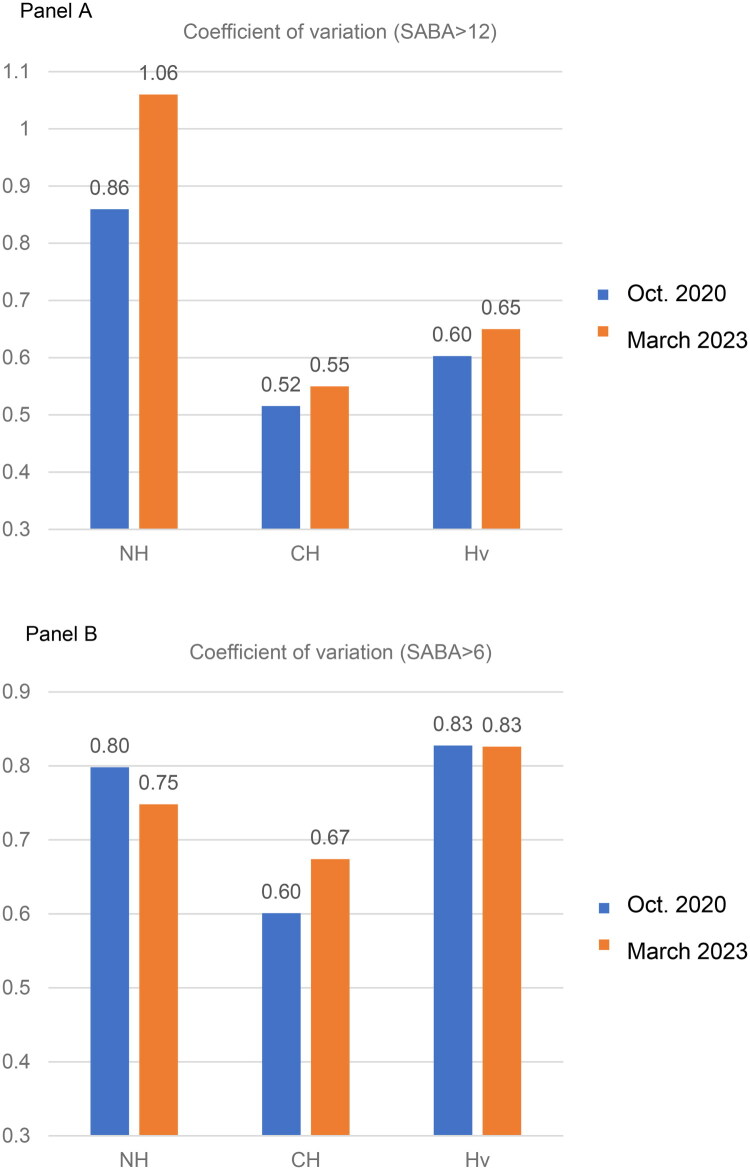
Bar charts plot illustrating the variability among practices of the proportion of patients with asthma prescribed ≥12 and ≥6 SABA inhalers in the previous 12 months, in the 3 CCGs. In blue, the variability values as in October 2020, in orange, the variability in March 2023. Higher coefficient means higher variability among practices in each CCG.

This variation in mean percentage of patients prescribed ≥12 SABA between practices was and stayed higher throughout the QI in Newham borough compared to the control boroughs, as shown in Figure 2, panel A. Indeed, we observed the mean percentage of patients prescribed ≥12 SABA inhalers/year in different practices belonging to the same PCN in Newham showed divergent trends, with some practices experiencing a decrease (e.g. from 11% to 2%) while others saw an increase (e.g. from 17% to 21%). In contrast, practices in City & Hackney exhibited lower variability in the mean percentage of patients prescribed ≥12 and ≥6 SABA inhalers (Figure 2, panel B), suggesting a more uniform uptake of new interventions, likely facilitated by stronger inter-practice relationships within the locality.

### Effects of COVID-19 pandemic

**Figure 3.**
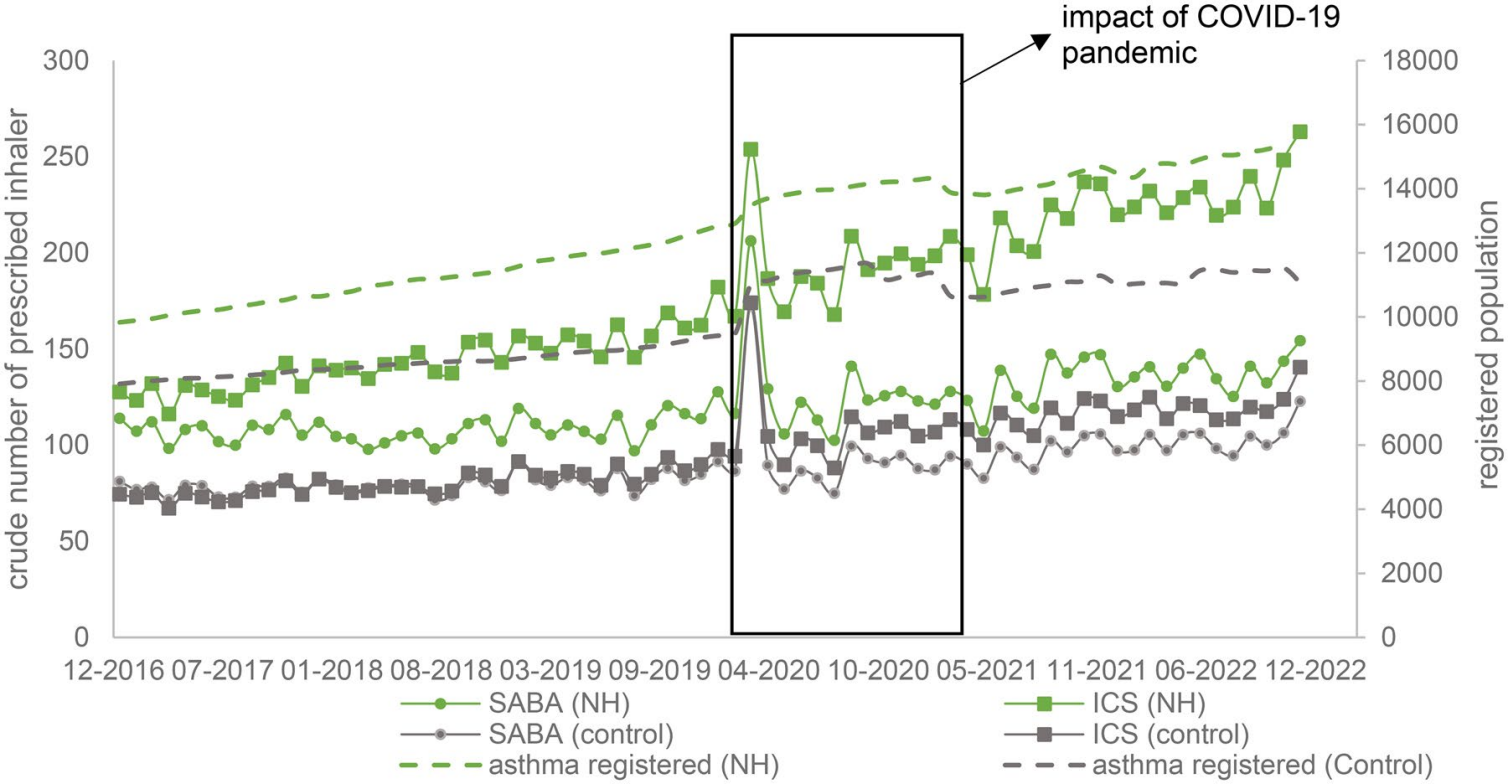
Pandemic effects on number of SABA and ICS prescriptions in NH and control group (CH and Hv).

The start of the COVID-19 pandemic in March 2020 was associated with a sudden, but temporary, increase in the number of patients on asthma registers. This was seen across both intervention and control boroughs (see Figure 3), This was associated with an increase in both SABA and ICS prescribing and persisted for about 6 months., affecting QI outcome measures.

### Effect of electronic alerts to SABA overprescription

**Figure 4.**
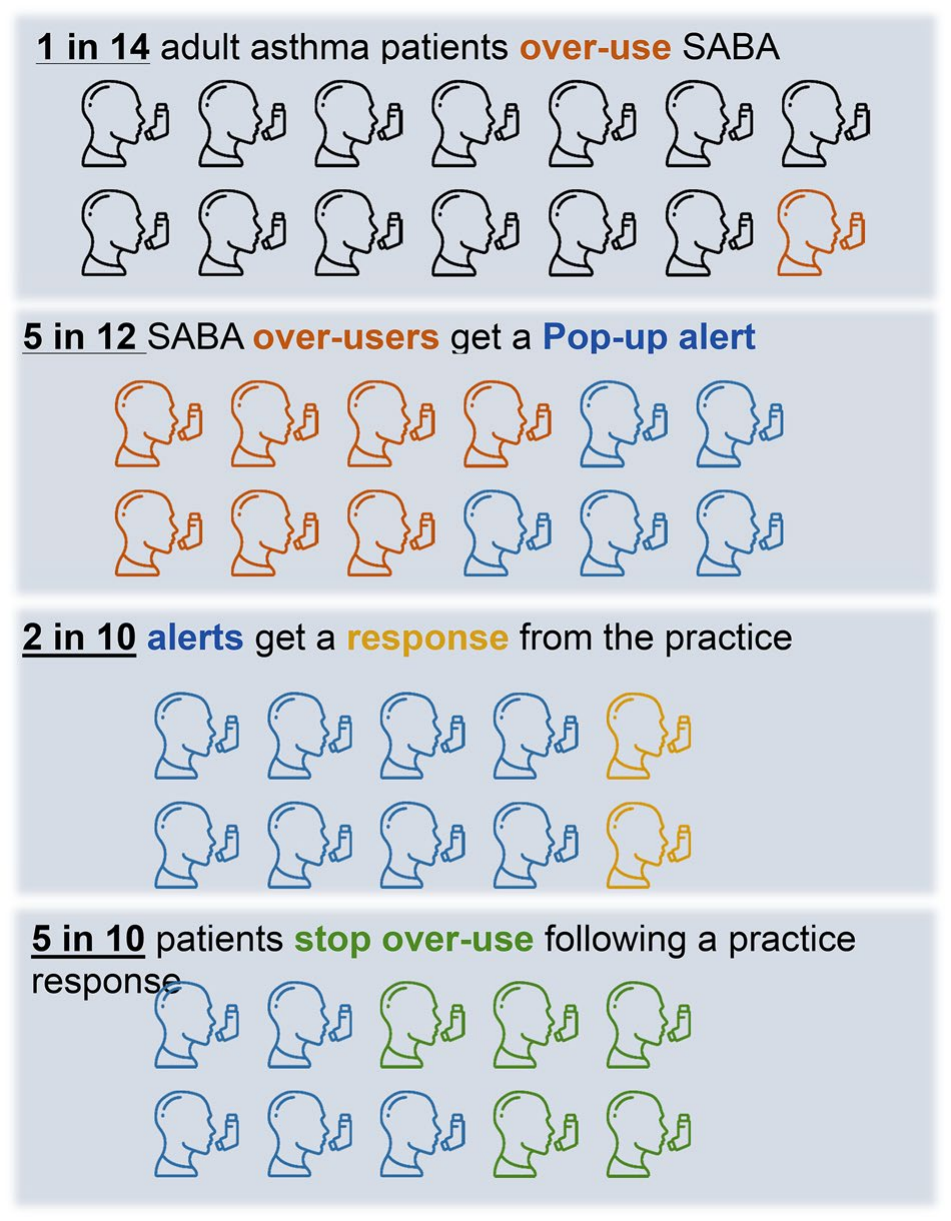
Infographic illustrating real-world effect of electronic alerts of SABA overprescribing.

The electronic alerts highlighting SABA overprescribing were acted upon in 17% of cases. Acting on the alert was associated with a successful decrease ibn SABA overprescribing: 50% of patients receiving an active response achieved a subsequent reduction in SABA overprescribing in the following year (see Table 1 and Figure 4).

**Table 1.** Total number of patients with an alert triggered in October and November 2020, April and May 2021, April and May 2022.

	Number	%
Patients issued more than 6 SABA in 6 months and an alert was triggered	2671	100
Patients with an alert, for whom an active response is recorded	442	17
Patients receiving an active response, and subsequent SABA use fell to <12 in the following year	221	44

We looked at activations in the intervention borough to see the breakdown of who viewed the alerts. There were 24,114 activations between October 2020 and April 2021, with 48% viewed by receptionists, admin, or managerial staff, 37% by GPs, 12% by pharmacists, and 3% by nurses.

An evaluation of the pop-up tool in one intervention practice showed a total of 881 activations. Of these, 52.2% were by receptionists, admin, or managerial staff, 35.3% by GPs, 9.9% by nursing or healthcare assistants, 2.5% by allied health professionals, and 0.1% by a medical student. In terms of specific actions prompted by the tool, there were 274 total activations leading to different outcomes. Of these, 90 activations led to a verbal invitation for an asthma review, 104 resulted in a medication review, and 80 generated a letter inviting the patient for an asthma review. The breakdown of these actions by role included 145 by GPs, 102 by reception/admin/managerial staff, 24 by nursing/HCAs, 2 by medical students, and 1 by an allied health professional.

### Feedback on the quality improvement from healthcare professionals

Feedback received *via* the survey [16] (*n* = 16 participants including GPs, nurses, pharmacists, and practice managers, see Supplementary material 2) was positive about the QI. Almost all were aware of the prescribing alerts and thought that the QI helped their practice to identify patients at high risk of hospital admission with asthma. However, they also noted that practice engagement was significantly impacted by the disruptions and increased workloads resulting from the COVID-19 pandemic.

Content analysis of notes taken during discussions with clinicians at webinars highlighted the need to test the tool in real time with staff during the webinars, which was subsequently implemented (see Box 2).

Topics which came up frequently in the webinars are identified in Box 2.

## Discussion

### Summary of main findings

This QI initiative employed a learning health system approach using electronic prescription data from primary care to test effective practice strategies for reducing SABA overprescribing and, consequently, asthma-related hospital admissions in East London. Practice engagement with the intervention arm of the programme varied, this was greatly affected by the disruption and altered workloads caused by the COVID-19 pandemic. Despite uneven engagement by practices, and the unknown effects of concurrent incentivised respiratory activities, our QI initiative contributed to a significant reduction in SABA over-prescribing compared to before the QI, and a steeper rise in ICS prescribing, though this did not reach significance in the study period.

This difference for Newham is equivalent to an absolute reduction of hospital admission of 11%, which is the estimated associated reduction in hospital admission comparing before and after the QI. This is based on data from a previous study on asthma prescribing, ethnicity and risk of hospital admission in East London [7], though it was not possible to disentangle the contribution of the different QI components and other concurrent SABA reduction initiatives.

The pop-up prescribing alerts integrated within the practice software were associated with a 50% reduction in overprescribing in the subsequent year. The value of these alerts was limited by the low level of practitioner engagement. However, these findings do reflect a real-world evaluation of this intervention, undertaken during the Covid pandemic, compared with a trial setting [18].

Qualitative insights from the online survey and practice staff attending the webinars support an effect of the QI activities, in particular reviewing the lists of patients with high SABA use helped to reduce the overuse of salbutamol and to identify the reasons behind it.

QI activities to reduce SABA overprescribing were affected by other local strategies (see Box 1), and by the strength of cohesion among local practices. Localities with stronger inter-practice relationships were able to implement clinical change more rapidly. These inter-practice factors may play an important role in QI implementation despite being poorly characterised.

The COVID-19 pandemic affected the QI programme in a number of ways. Practice staff were distracted with new ways of working, and were often unable to gain effective mentoring in the use of electronic tools. The respiratory symptoms of COVID triggered a surge in patients reactivating an asthma diagnosis, and requesting SABA inhalers.

### Comparison with existing literature

These results confirm in the real-world setting of primary care, the previous systematic review evidence that electronic alerts can reduce excessive prescribing of SABA, when delivered as part of a multicomponent intervention [19]. This quality improvement program highlights the strength of a collaborative, comprehensive model to support patients with asthma overprescribed SABA [11,12,19]. The SENTINEL programme pilot [20] was implemented contemporaneously with our QI and similarly resulted in reductions in SABA prescribing.

The distinctions between the two approaches warrant clarification. Whereas the primary focus of our QI programme was the reduction of SABA overuse, start or optimisation of ICS, adherence support and offer of educational resources, SENTINEL concentrated on transitioning patients from SABA to combined maintenance and reliever therapy (MART). In SENTINEL, patient prioritisation for clinical review was based on SABA overuse in the preceding 12 months, with those using ≥6 inhalers reviewed first. By contrast, our programme targeted individuals with a combination of high SABA use and low ICS use, reflecting a broader assessment of suboptimal controller therapy, and used in addition an electronic alert approach to identify patients overprescribed SABA. It is also important to highlight that local clinical guidelines did not adopt AIR/MART as first-line therapy until 2023, i.e. after completion of this QI. This QI was conducted ahead of national UK guidelines changing in November 2024.

### Strengths and limitations

The study results are based on 16 thousand patients with asthma from a population of almost one million GP-registered patients in East London. All practices in the intervention borough showed some engagement with the QI programme. By using a full year of SABA prescribing as outcome data, the expected fall in SABA use was only expected to begin at least one year after the start of the interventions. The COVID-19 pandemic disrupted the programme, increasing workload and limiting the attention practices could give to the project. No attempts were made to analyse the characteristics of patients who decreased their use of SABA following the activation of the prescribing alert.

Control boroughs exhibited stronger declines before and during the intervention, whereas the intervention borough decline occurred mainly towards the end. This delayed effect is likely explained by several contextual factors. Many intervention practices initially required webinar-based support to access and interpret their prescribing data and to understand the aims of the QI, meaning substantive changes may have been implemented only later in the period. Furthermore, the intervention coincided with significant COVID-19–related operational pressures, including the shift to remote consultations and remotely delivered education, which likely slowed early implementation and contributed to the differing temporal patterns between groups.

A limitation of the results from the interrupted time series analysis is that the trend in SABA prescribing was not stable, as it was already exhibiting a decreasing trajectory prior to the onset of the QI initiative, potentially confounding the assessment of its true impact.

While the initiative was limited to primary care within a specific region of London, it highlights the crucial need for a whole-system approach [21].

The study measured prescriptions issued rather than medication taken. Receiving a prescription does not necessarily mean that the prescription was filled and used, and it is possible that ‘stock piling’ inhalers may inflate estimates of SABA overprescribing. This will also contribute to medication waste.

A substantial proportion of activations (52.2%) were seen by receptionists and admin staff. Because these activations were not reviewed by clinicians, opportunities for timely correction, reinforcement, or intervention may have been missed.

### Implications for practice and research

Our results emphasise the importance of taking patients off repeat, and repeat-dispensed prescriptions for inhaled asthma medications as previously described [9]. Engagement with electronic prescribing alerts, by clinicians, and also by administrative staff, is an effective intervention to decrease the number of patients overprescribed SABA. This study also highlights the importance of practice teams working effectively with pharmacists to build a shared understanding of managing access to SABA medications. Extending pharmacist roles into patient reviews can also make effective contributions to reduce SABA overprescribing.

In the near future practices will switch many adults to MART. Future research could focus on investigating the effect of switching to MART on SABA prescribing, and the effect on hospital admission rates, given that such changes are already occurring in practice. These findings would have important implications for clinical care, as a broader transition to MART is likely to reduce unnecessary SABA use and meaningfully lower hospital admissions, reinforcing the urgency of adopting MART as a standard approach where appropriate.

## Supplementary Material

Supplemental Material

## Data Availability

All data relevant to the study are included in the article. Other local quality improvement activities and initiatives potentially affecting the intervention. **Quality improvement activities** We ran 21 webinars within Newham PCN and practice meetings, nurse and pharmacist forums. They were attended by 326 staff in total, and included primary care practitioners, pharmacists, practice managers and admin staff. 16/21 webinars included practice staff sharing screens and being coached to download a run the EMIS tool, generating the list of patients overprescribed SABA. Since mid-2021 webinars were mainly run with pharmacists working in local practices. The YouTube video [18] attracted 259 views and the SurveyMonkey feedback form was filled by 16 participants (4 GPs, 4 nurses, 2 practice managers, 4 pharmacists, 1 admin). **Concurrent asthma activities affecting the quality improvement** During the three years of the QI, all practices in the study area undertook a range of incentivised activities connected with limiting excessive SABA prescribing. From 2015 City & Hackney commissioned Practice Support Pharmacists (PSPs), who are experienced pharmacists dedicating time to implement agreed guidelines through patient reviews, prescribing audits, education and training at practice level, working with the local medicine management team (MMT). From April 2021 to March 2022, Havering borough, as part of Barking Havering and Redbridge (BHR), was the recipient of an Incentivised Respiratory Scheme aimed at reducing the number of prescribed inhalers in asthma and COPD (Long Term Condition (LTC) Group 2 Localised Incentive Scheme (LIS)). A previous LIS three years earlier in 2019 standardised the asthma review process with medication reviews to reduce SABA overprescribing. From July 2021 to March 2022 Newham borough was part of an incentivised Medicine Optimisation Scheme, which included reducing SABA overprescribing in asthma and COPD. Summary of comments raised at webinars. **Tips for clinicians**
Inform newly registered patients with asthma that SABA is not available as repeat prescription. Involve practice pharmacists in reviewing lists of patients who overuse SABA. **Barriers to using the quality improvement tools**
Coaching required for practice team members to use the electronic tools Administrative staff need training to respond to in-consultation prescribing alerts. **Problems in managing SABA overprescription**
Patient complaints when GPs decline to prescribe. Pharmacists describe patients ‘borrowing’ inhalers Patients attending multiple pharmacies for ‘emergency SABA’. Overprescribing related to repeat dispensing.
