# Supplementary material for: Reducing short-acting beta-agonist overprescribing in general practice: Evaluation of a quality improvement programme in East London
Source: Eur J Gen Pract. 2026 Feb 2;32(1):2619229. doi: 10.1080/13814788.2026.2619229 (PMC12865841; doi:10.1080/13814788.2026.2619229)
Supplement: Supplemental Material [file IGEN_A_2619229_SM2003.pdf]

SUMMARY GUIDELINES

JANUARY 2020

Identifying patients with asthma at high risk of hospital admission

Primary Objectives

Improve identification of patients who are at higher risk of hospital admission due to their asthma.

Provide practices with EMIS IT tools to support the identification of patients with high risk asthma based on prescribing records.

Optimise asthma management and prescribing with regular structured asthma reviews.

The problem

The UK has among the highest rates of asthma, and asthma deaths in Europe.

Asthma accounts for 60,000 hospital admissions a year.[3]

In East London, hospitalisation is 14% above the average for London.[4]

In the UK About 1,200 people a year die from asthma.[5]

Two in three of these deaths could be prevented by better management.[6]

Key points in asthma management

A structured clinical review

Patients with asthma should have a structured clinical review at least annually. This should include:

Symptoms and triggers

Inhaler technique

Update of a personalised self-management plan

A regular prescribing review:

A well-controlled asthmatic patient will require their salbutamol no more than twice weekly (equivalent to one device per year).

Use of more than one SABA inhaler device a month suggests poor asthma control, and requires early review.

Adequate use of inhaled corticosteroid treatment should be assessed.

Long-acting inhaled β2 agonists (LABA) should only be used in patients on ICS. Use a combined ICS/LABA to improve safety.

For detailed advice on prescribing see BTS/SIGN guidlines[1] and the local CCG guidelines for management of asthma in adults.[2]

REAL-HEALTH RESPIRATORY

BARTS CHARITY

ceg Clinical Effectiveness Group

Barts and The London School of Medicine and Dentistry

Queen Mary University of London

NHS

Current risk prediction models

A review of published risk prediction models found that three factors were predictors of future exacerbations:

a. previous health care utilisation
b. symptoms
c. spirometry values.[8]

Access to secondary care data and good coding of exacerbations and AED/hospital admission are required for these models.

SABA overuse is a simple and reliable proxy for symptoms and spirometry values.

Our systematic review[9] confirms that electronic alerts reduce excessive prescribing of SABAs, when delivered as part of a multicomponent intervention.

Asthma UK in conjunction with EMIS Web released a prescribing alert to highlight patients using excess SABA. This alert will activate if there are 3 prescriptions for SABA within a 3-month episode. *However, this assumes that only one device is issued per prescription and may underestimate SABA overuse.*

East London Asthma study

A local study[7] of asthma linking GP and hospital records showed that:

13% of adults with asthma were prescribed >12 SABA inhalers a year.

Three quarters of adults with asthma use ICS. But 76% were prescribed fewer than 10 ICS canisters a year (underuse).

Hospital admission rises from 1.3 to 7.5 per 100 asthma population as the number of SABA inhalers prescribed rises from 1–3 to 13+ a year.

Rate of hospital admission for adults with asthma using ICS, stratified by SABA use[7]

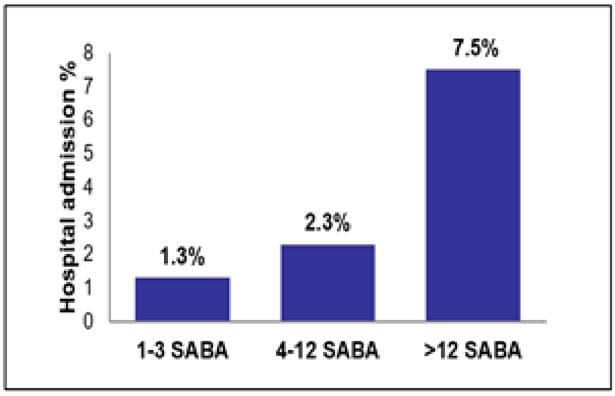

Two new CEG tools to help identify patients with asthma at risk of hospital admission.

1. Pop-up SABA alert

The tool counts the SABA medication (salbutamol but not terbutaline) issued in the previous five months. It calculates the numbers of inhalers (rather than the number of prescriptions), therefore provides an accurate measure of SABA use prescribed by the practice. This tool overcomes the limitation of the EMIS-Asthma UK on-screen message triggered when more than 3 SABA are issued in the last 3 months. (Which assumes one inhaler per prescription)

The new pop-up alert tool displays at the centre of the screen. The clinician has to dismiss it before proceeding. The alert is designed to open when the medical record is opened and triggers only once on any day.

The user is invited to take further action from a selection of options which will depend on whether the patient is with the clinician at the time.

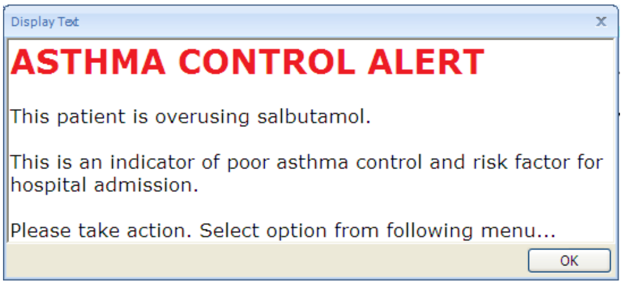

The second option (see next page) includes automatically generating a personalised letter to the patient inviting them to make an appointment for review.

Using the Pop up alert in your practice

The alert is designed to be used by GPs, practice nurses, other clinical staff such as pharmacists and administrative staff. Only GPs are able to permanently deactivate the alert.

Each option within the alert is associated with a Read code. Hence it is possible to audit the alert activations and the actions taken by different users within the practice.

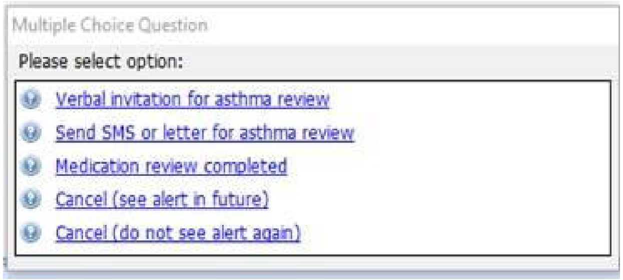

2. Asthma prescribing trigger tool

- The asthma prescribing trigger tool is used at practice level to identify patients who are at higher than average risk of asthma attacks and hospital admission and arrange for them to have a clinical review.

- This technique has been used successfully in Tower Hamlets for identifying at risk patients with anticoagulation in atrial fibrillation, hypoglycaemia in type 2 diabetes and a falling eGFR in chronic kidney disease.

- The process consists of two parts:

1) A practice based EMIS search which exports predefined patient data from the clinical system.

2) An Excel file which imports this data for analysis.

The Excel file can stratify patients based on their inhaler use, identifying patients for early review to clinical and administrative teams.

Every practice will be sent a guide to using the asthma trigger tool. CEG facilitators will support the practice use of the tool.

The asthma trigger tool is designed to be run on a regular basis within the practice – monthly or quarterly. It can be used to identify those most in need of asthma management and prescribing review. The space for clinical reflection allows documentation of the planned intervention for that patient. The completed sheets can be used as audits of asthma review and management for appraisal.

The figure below shows the practice facing Excel sheet, with the patient list ordered by salbutamol dose in the previous 12 months, with the highest number at the top. The tool also lists the number of ICS inhalers (but does not take account of different strengths of ICS devices). The filters allow the user to find the patients in most urgent need of review because they have the highest SABA and lowest ICS use.

The SABA Alert and Trigger Tool only capture one risk indicator for poor asthma control. Previous exacerbations requiring oral corticosteroids (prednisolone) and previous healthcare usage for acute asthma are also markers of poor asthma control. Biomarkers (blood eosinophil counts and FeNO tests) also have a role in estimating exacerbation risk, though availability in primary care is currently limited.

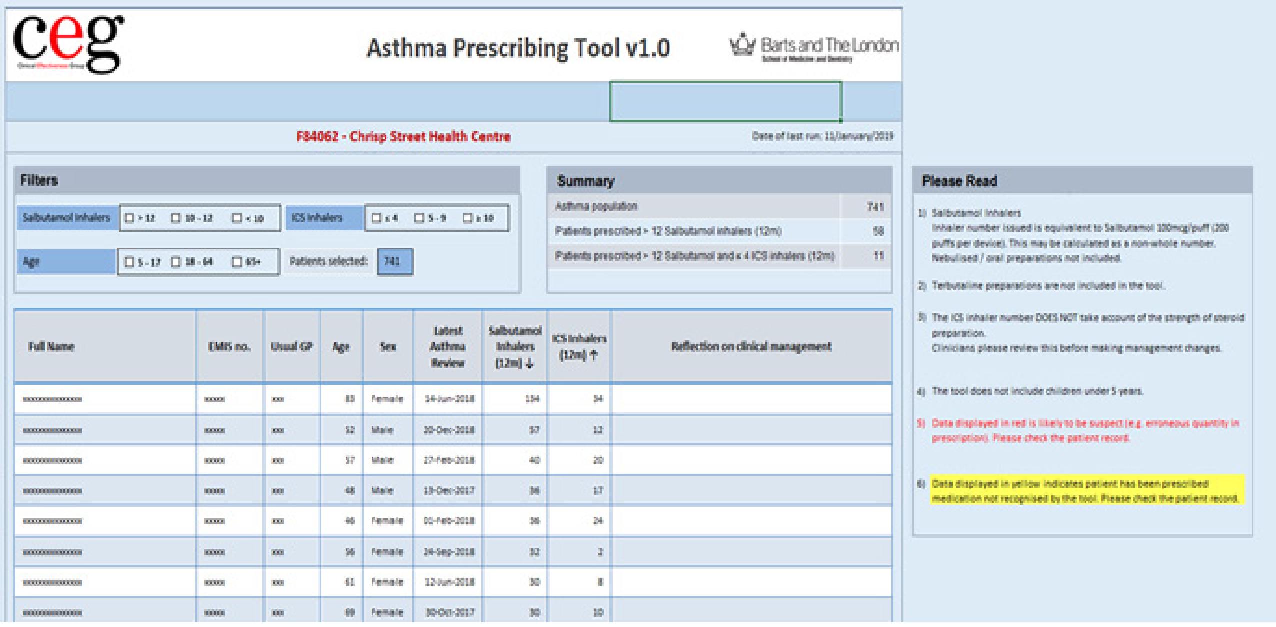

Full Name	EMIS no.	Usual GP	Age	Sex	Latest Asthma Review	Salbutamol Inhalers (12m) ↓	ICS Inhalers (12m) ↑	Reflection on clinical management
xxxxxxxxxxxxxx	xxxxx	xxx	83	Female	14-Jun-2018	134	34	
xxxxxxxxxxxxxx	xxxxx	xxx	52	Male	20-Dec-2018	57	12	
xxxxxxxxxxxxxx	xxxxx	xxx	57	Male	27-Feb-2018	40	20	
xxxxxxxxxxxxxx	xxxxx	xxx	48	Male	13-Dec-2017	36	17	
xxxxxxxxxxxxxx	xxxxx	xxx	46	Female	01-Feb-2018	36	24	
xxxxxxxxxxxxxx	xxxxx	xxx	56	Female	24-Sep-2018	32	2	
xxxxxxxxxxxxxx	xxxxx	xxx	61	Female	12-Jun-2018	30	8	
xxxxxxxxxxxxxx	xxxxx	xxx	69	Female	30-Oct-2017	30	10	

Measuring Impact

As part of the three year REAL-HEALTH respiratory programme we aim to evaluate the impact these practice tools have on rates of SABA overuse and asthma exacerbations requiring hospital admission.

This REAL HEALTH programme is funded by Barts Charity.

References

1. BTS/SIGN guidelines.

2. Barts Health Trust - Guidelines for the Management of Asthma in Adults, 2018. https://www.nelft.nhs.uk/download.cf-m?doc=docm93jijm4n3772.pdf&ver=6237

3. WHO Europe Hospital Morbidity Database (HMDB).

4. East London data: The Tower Hamlets Joint Strategic Needs Assessment Reference Group 2015

5. Office for National Statistics for England and Wales, the General Register Office for Scotland and the Northern Ireland Statistics and Research Agency.

6. The National Review of Asthma Deaths, 2014.

7. Hull S et al. NPJ Prim Care Respir Med. 2016; 26: 16049.

8. Loymans et al. J Allergy Clin Immunol Pract. 2018;6(6):1942-1952.e15.

9. McKibben S et al. NPJ Prim Care Respir Med 2018 16;28(1):14.

Authors

This guideline was written by Anna De Simoni, Sally Hull, Chris Griffiths, Paul Pfeffer and Jim Cole. Unless stated otherwise it conforms to national NICE guidance.

This summary guideline does not replace clinical judgement in individual circumstances.

This guideline and more information about the REAL-HEALTH programme are available on the CEG website: www.qmul.ac.uk/blizard/ceg/

Clinical Effectiveness Group
Centre for Primary Care and Public Health
Barts and The London School of Medicine and Dentistry
58 Turner Street
London E1 2AB
Tel: 020 7882 2553
email: ihse-ceg-admin@qmul.ac.uk

Queen Mary University of London

Barts and The London School of Medicine and Dentistry

Identifying patients with asthma at high risk of hospital admission

KEY RECOMMENDATIONS

- Use the *in-consultation* EMIS pop-up and the *back office* asthma prescribing trigger tool to identify high-risk asthmatics
- Consider using our suggested asthma control questions in your annual review and our management flowchart to optimise asthma treatment
- Provide your patients with locally adapted asthma educational resources

SUGGESTED NEXT STEPS

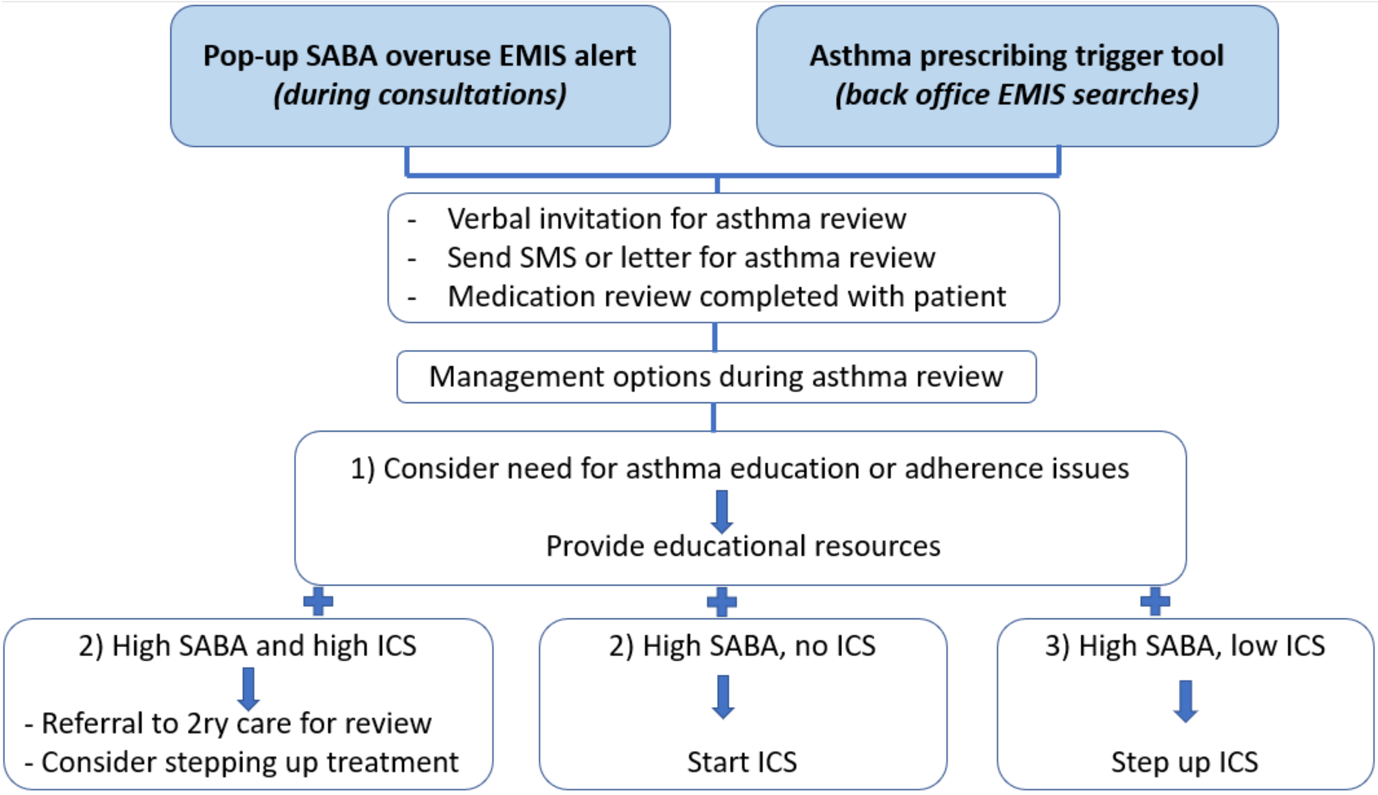

1) **Asthma education needs or adherence issues:** see next page for resources.

2) **Patients overusing SABA and on high dose ICS:** may have a poor response to treatment, unclear diagnosis, or suspected occupational asthma. ***Consider Referral to secondary care for review***.

3) **Patients overusing SABA and not on ICS:** Start ICS. 400 micrograms/day is an appropriate starting dose.

4) **Patients overusing SABA *on-low dose ICS***: Add ***inhaled long-acting β2 agonist (LABA).***
   a. If there is benefit from LABA but control is still inadequate: ***continue LABA and increase inhaled corticosteroid dose to 800 micrograms/day.***
   b. No response to LABA: ***stop LABA and increase inhaled corticosteroid to 800 micrograms/day.***
   c. If control still inadequate: ***consider leukotriene receptor antagonist or SR theophylline.***

ASTHMA EDUCATIONAL NEEDS OR ADHERENCE ISSUES

- Provide patient handout of educational resources (linked to template)

EDUCATIONAL RESOURCES

- Mobile apps: freely available to download: RightBreathe.
- Videos: Inhaler technique and using the spacer: https://www.asthma.org.uk/advice/inhaler-videos/
- Local group activities: Breathe Easy or singing groups: https://www.blf.org.uk/support-in-your-area
- Social Prescribing: https://www.london.gov.uk/what-we-do/health/social-prescribing

SUGGESTIONS FOR QUESTIONS TO ASK DURING ASTHMA REVIEWS

- **Occupational asthma**
  Did your asthma symptoms start as an adult, or have your childhood asthma symptoms returned since you started working?
  Do your symptoms improve on the days you're not at work, or on holiday?
  Do your symptoms get worse after work or disturb your sleep after work?

- **Presence of rhinitis (maybe linked to poor asthma control)**
  Do you suffer from two or more of the following symptoms regularly: runny nose, sneezing, nasal congestion, itching and red, watery eyes.

- **Asthma triggers**
  Which asthma triggers affect you? Alcohol, Animals and pets, Viral URTIs, Emotions, Exercise, Food, Female hormonal changes, Dust mites, Indoor environment, Moulds and Fungi, Pollen, Pollution, Recreational drugs, Sexual activity, Cigarette smoke, Stress and anxiety, Cold weather.

- **Stigma related to asthma**
  Research shows that there is stigma associated with asthma. Patients may be embarrassed about the diagnosis and having to use an inhaler. Have you ever experienced this?

Sometimes people appear to be overusing SABA, but may just be 'stocking up' with an inhaler to keep at work, at school or in the car.

Funded as part of the REAL HEALTH programme supported by Barts Charity.

Clinical Effectiveness Group, Centre for Primary Care and Mental Health,
Barts and the London Medical School, Yvonne Carter Building, 58 Turner Street, London E1 2AB
Phone: 020 7882 2553, Website: *http://www.blizard.qmul.ac.uk/ceg-home.html*

Patient information sheet **January 2020**

How do I look after my asthma?

Looking after your asthma and health can be challenging.
Please find below suggestions about self-managing your asthma

1. **Mobile apps to get to know better your inhalers**
   freely available to download: RightBreathe
   Google 'right breathe' or find the app Rightbreathe on your mobile Google Play or Apple App store

2. **Videos to check your inhaler technique and your use of a spacer**
   Google 'Asthma UK inhaler-videos 3 minutes'

3. **Did you know about local group activities organised by the British Lung Foundation?**
   Join Breathe easy or singing groups
   Google 'blf and local support' to find where, when and how to join them

4. **Have you seen the Asthma UK resources for adults with asthma?**
   Google 'Asthma UK resources'.
   You can download:
   1. An asthma action plan
   2. 'Live well with asthma' booklet
   3. 'Make the most of your asthma review' booklet
   4. A peak flow diary

   You can also connect with other 13,000 users on the Asthma UK discussion forum and. Join a discussion or start your own on the forum!

Funded as part of the REAL Health programme supported by Barts Charity.

BARTS CHARITY
